# Mechanism Analysis and Self-Adaptive RBFNN Based Hybrid Soft Sensor Model in Energy Production Process: A Case Study

**DOI:** 10.3390/s22041333

**Published:** 2022-02-10

**Authors:** Junrong Du, Jian Zhang, Laishun Yang, Xuzhi Li, Lili Guo, Lei Song

**Affiliations:** 1 Key Laboratory of Space Utilization, Technology and Engineering Center for Space Utilization, Chinese Academy of Sciences, Beijing 100094, China; dujunrong18@csu.ac.cn (J.D.); zj@csu.ac.cn (J.Z.); xzhli@csu.ac.cn (X.L.); guolili@csu.ac.cn (L.G.); 2 School of Computer and Technology, University of Chinese Academy of Sciences, Beijing 100049, China; 3 College of Civil Engineering and Architecture, Shandong University of Science and Technology, Qingdao 266590, China; yangls@sdust.edu.cn

**Keywords:** soft sensor, coal mill ventilation, mechanism analysis, radial basis function neural network, genetic algorithm

## Abstract

Despite hard sensors can be easily used in various condition monitoring of energy production process, soft sensors are confined to some specific scenarios due to difficulty installation requirements and complex work conditions. However, industrial process may refer to complex control and operation, the extraction of relevant information from abundant sensors data may be challenging, and description of complicated process data patterns is also becoming a hot topic in soft-sensor development. In this paper, a hybrid soft sensor model based mechanism analysis and data-driven is proposed, and ventilation sensing of coal mill in a power plant is conducted as a case study. Firstly, mechanism model of ventilation is established via mass and energy conservation law, and object-relevant features are identified as the inputs of data-driven method. Secondly, radial basis function neural network (RBFNN) is used for soft sensor modeling, and genetic algorithm (GA) is adopted for quick and accurate determination of the RBFNN hyper-parameters, thus self-adaptive RBFNN (SA-RBFNN) is proposed to improve the soft sensor performance in energy production process. Finally, effectiveness of the proposed method is verified on a real-world power plant dataset, taking coal mill ventilation soft sensing as a case study.

## 1. Introduction

Energy production process is important to ensure national economic development and resident’s lives quality [1]. To achieve real-time control, operation optimization and process prediction, a significant number of hardware sensors are placed to supply data for intelligent monitoring [2]. As a result, cost and difficulty of sensors installation and debugging for vital parameters are increasing [3]. However, due to wicked installation demands and complex work conditions in industrial process, hard sensor is limited to obtain the fast and accurate sensing result. Recently, soft sensors have been widely used for online estimation of process parameters thanks to their rapid response, low maintenance costs, and accurate prediction. Soft sensors can predict the difficult-to-measure parameters by building predictive mathematical models based on hard sensor process variables that are easy to measure [4,5,6].

Basically, soft sensor models are divided into two main categories, model-driven and data-driven methods [3]. Model-driven method, also known as white box model, employs first principle modeling based on system’s physical knowledge. This kind of approach can work well if detailed and accurate mechanism model or a wealthy of priori knowledge about process is available. However, increasing complexity of industrial process makes these preconditions difficult to meet. On the contrary, data-driven method, considered as black box model, relies on information extraction and regression training from system’s historical data. Wide arrays of machine learning techniques are developed solely using available data without necessary physical meaning of the resulting models [7]. Nevertheless, energy production process always has complex nonlinear behavior due to complicated physiochemical mechanism, working condition and dynamic noise, and it is challenging for feature representation and data pattern learning from high dimensionality of process data [8].

Given common use of energy production systems and relative availability of historical process data, data-driven soft sensors have become popular [8]. Typical data-driven models are principal component regression (PCR) [9], partial least squares [10], and artificial neural network (ANN) [11] etc. Zheng [12] develops a feedforward multilayer perceptron (MLP) based ANN model to estimate the apparent viscosity of the water-based drilling fluids, and the results show the neuron model has better performance than statistical models. Wang [13] proposed an improved particle swarm optimization (PSO) algorithm is embedded into SVM to enhance the soft sensor accuracy of net calorific value of coal, and the compared results show the improved PSO-SVM is superior to support vector machine (SVM), Long Short-Term Memory (LSTM) and Backpropagation neural network. A just-in-time learning (JITL) based mixed kernel principal component weight regression method is proposed for industrial soft sensing, mixed kernel principal component analysis is developed to extract nonlinear features and a weighted regression based just-in-time learning strategy aims to deal the time-variant problem of processes [14]. These classical machine learning methods can be regarded as shallow learning networks, and they are limited in capturing complicated data features since the process data are growing with high dimensionality and large volume [15].

In the last decade, deep learning techniques have become an alternative for feature representation and pattern learning in different complex soft sensor tasks. Due to the stacked structure of multiple layers of deep architecture, deep NNs are able to represent any highly nonlinear data relationships [3,16]. By far, deep learning has achieved breakthrough results and outperformed many state-of-the-art methods, such as SVM [17]. Since hierarchical deep features are learned for raw input data progressively, deep learning is more suitable and powerful to discover intricate data patterns [18]. Deep belief network (DBN) [19], LSTM [20], Convolutional Neural Network (CNN) [21], and Stacked Autoencoder (SAE) [22] are some of the most popular deep learning models that have performed successfully in complex modeling tasks. In [23], two different models are conducted to soft sensing the industrial noxious gas, experiment demonstrates DBN based soft sensor method is more robust with less hyper-parameter than PCA based multilayer perceptron neural network. A novel soft sensor model combining Xgboost decision trees and Bi-directional LSTM is proposed, the overfitting problem is successfully averted and the effectiveness of the proposed method is verified [24]. To address the problem of the loss of the intrinsic geometric structure embedded in raw data, a nonlocal and local structure preserving stacked autoencoder (NLSP-SAE) is proposed for soft sensor, and experiment demonstrates NLSP-SAE can improve the prediction accuracy of quality variables [25]. Though hierarchical features can be learned from industrial process data via unsupervised pretraining, and learned features can represent original data well. However, unsupervised pretraining often neglects important object-relevant information to guide feature learning, and learned features that are lack of interpretability may contain irrelevant information for soft sensor [15]. Moreover, as online application demand, computational efficiency is also a challenging issue for deep learning based soft sensing.

To alleviate the problems above, this article proposes a hybrid model combining mechanism analysis and self-adaptive RBF neural network for soft sensor modeling in energy production process. To the best of our knowledge, hybrid driven approach is seldom studied and applied in soft sensor fields. Taking coal mill ventilation (CMV) sensing in power plant as a case study, the proposed methodology is verified effectively for soft sensor modeling. The main contributions of the work are described as below:

Firstly, a novel hybrid soft sensor model combining mechanism analysis and data-driven is proposed, the former is used to identify strongly relevant-features for soft sensor and the latter aims to learn highly nonlinear relationships from high dimensionality from process data.

Secondly, RBFNN is used as a basic model for soft sensor, while GA algorithm is adopted to acquire a new empirical formula for quick and accurate determination of hype-parameters of RBFNN. Thus, the accuracy and computational efficiency of soft sensor are extremely improved.

Finally, experiment on a real power plant dataset is conducted, and results demonstrate that the proposed model performs the best in terms of accuracy and computational efficiency, comparing with the classical methods.

The rest of this article is structured as follows. Firstly, theoretical principles of the used algorithms are described in Section 2. Then Section 3 shows us the overview of the proposed methodology, including the technical process of the proposed method and the evaluation indicators of soft sensor models. Finally, experiments on a real-world dataset from a power plant is conducted for validation of the proposed methodology, and Section 5 the conclusions of this study is made.

## 2. Theoretical Background

### 2.1. Radial Basis Function Neural Network (RBFNN)

**Figure 1:**
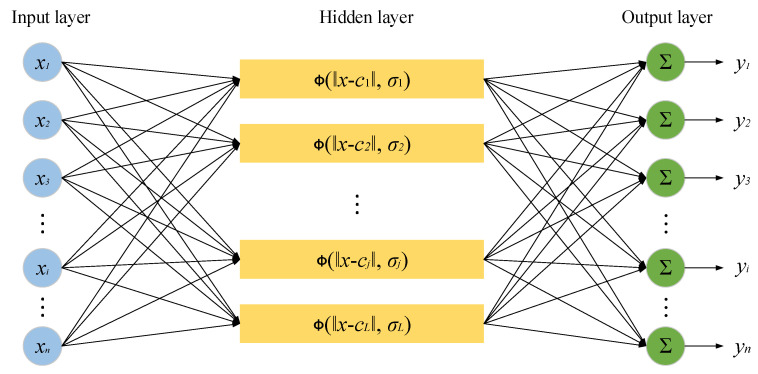
The structure of radial basis function neural network.

Radial basis function (RBF) is a kind of function, of which the value depends on the actual distance from the origin. RBF was first proposed by Powell and has been widely used in the analysis of clustering and pattern recognition [26]. RBFNN is an artificial neural network with RBF as an activation function. A typical RBFNN consists of three layers: input layer, hidden layer and output layer, shown in Figure 1. Input layer receives input variables and pass them into hidden layer. Hidden layer adopts RBF as a base function, and maps input variables into the hidden space. Actually, as the base function of hidden layer, RBF works via the theory of kernel function and maps input vector from low dimensional space into high dimensional space, increasing the identifiability of input vector. Furthermore, the output of RBFNN is the linear weighted sum of output layer. Therefore, RBFNN realizes the nonlinear mapping between input vectors and output vectors, and the adjustable parameters of output layer are linear, greatly speeding up the learning rate and avoiding local minimum problem.

Due to strong global mapping capability, RNFNN is considered as one of the best feedforward neural networks with good performance in classification and regression tasks [27,28,29]. Taking the input vector *x* as an example, the output of RBFNN is defined as follows.

(1) $$y_{i}(x)=w_{0i}+\sum_{j=1}^{L}w_{ji}\Phi (\left\|x-c_{j}\right\|,\sigma_{j})$$

where, *x* is the input of RBFNN, *w_ji_* is the weight connecting the *j*th hidden layer node to the *i*th output layer node, *c_j_* is the center of the *j*th hidden node, *σ_j_* is the spread factor i.e., the activation degree of the *j*th radial basis function. *w_oi_* is the bias of the *i*th output layer node, and ||·|| denotes the Euclidean norm. Φ is the activation function of RBFNN, and Gaussian function [2] is the most commonly used function, of which the formula is described below.

(2) $$\Phi =G(x,x_{i})=G(\left|x-x_{i}\right|)=\exp(-\frac{1}{2\sigma_{i}^{2}}{\left\|x-x_{i}\right\|}^{2})$$

where, *x_i_* denotes the center of the function, *σ_i_* is the Gaussian function deviation that determine the response degree of activation function to input.

The variable hyper-parameters of RBFNN are the centers of RBFs, the spread factor *σ* of RBF and the number of hidden layers. Every RBF center represents an obvious input pattern which is completely different from the other modes, and the simplest method to specify RBF centers is randomly select a subset of inputs from training set. However, comparing to random selection method, unsupervised self-organizing approach is a more utilized and effective method. Actually, clustering is a data-analysis tool for characterizing the distribution of a dataset, and is always used for determining RBF centers [30]. In this paper, training set is grouped into appropriate clusters by k-means algorithm. The number of clusters, which is equal to the number of hidden units in RBFNN, can be specified via unsupervised training, and the clusters centers can be assigned as the centers of RBFNN. 

Furthermore, the spread factor *σ* which corresponds to the standard deviation of Gaussian function represents the activation degree of activation function [31]. The spread factor of Gaussian kernel based RBFNN is usually determined via two methods: one is random initialized method (RI-RBFNN) and the other is empirical formula method (EF-RBFNN). Therein, empirical formula for determining the spread factor *σ* is described below.

(3) $$\sigma =\frac{d_{\max}}{\sqrt{2L}}$$

(4) $$d_{\max}=\underset{i,j=1,\cdots ,L;j>i}{\max}\left|c_{i}-c_{j}\right|$$

where *d*_max_ is the maximum distance between the RBF centers, *L* is the number of hidden units, *c_i_* and *c_j_* are respectively the *i*th and *j*th center of the hidden node. It proves that this method can make Gaussian kernel based RBF neither too steep nor too flat. Thus, the spread factors for all Gaussian RBF centers can be calculated via Equations (3) and (4).

### 2.2. Improved Genetic Algorithm (GA)

**Figure 2:**
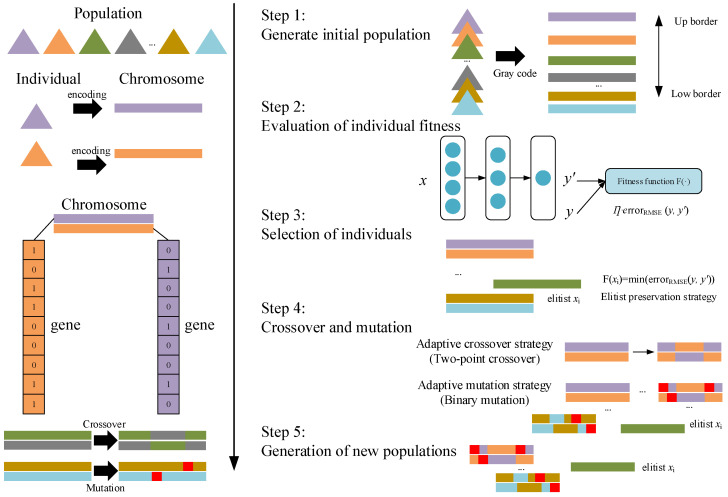
The schematic diagram of improved GA algorithm.

GA is a heuristic-based optimization technique that is inspired by Charles Darwin’s theory of natural evolution, and it is skilled in finding optimal or near-optimal solutions for difficult problems [32]. GA is a widely applied global optimization algorithm [33,34], but the primitive GA usually has some convergence and local optimality problems. Thus, we adopt elite preservation strategy and adaptation strategy. In primitive GA, x is referred as an individual, and each individual consists of chromosomes, which are expressed as a sequence of variables, or a set of genes, and multiple individuals are aggregated to form a population. The detailed technical process and schematic diagram of improved GA are shown below in Figure 2.

- Step 1: Generating the initial population

The chromosomes of individual x are first encoded into simple strings or number strings, followed by an algorithm that generates a certain number of individuals at random, and sometimes the operator can intervene in random generation process to improve the quality of the initial population. The usual encoding methods are binary encoding, tree encoding and Gray code. In this paper, the Gray code encoding method is chosen to encode the individuals, and the up and low bounds of the generated individuals are specified to intervene in the initial population generation conditions, as shown in Step 1 of Figure 2.

- Step 2: Evaluating the fitness of individuals

Adaptability is the ability of individuals in a population to adapt to the environment, that is, how well individuals perform in their environment. The calculation of fitness is also a crucial issue in the use of genetic algorithms. It specifies the direction of the search for a solution to the problem and is directly related to the efficiency of the search and the quality of the final solution. The fitness value is usually derived from the objective function. In order to cope with the two opposing optimization objectives of minimization and maximization, the minimization convention is generally followed, i.e., “the larger the value of the objective function, the smaller the fitness”. In this paper, for the calculation of the fitness, we use the calculation function of the evaluation metric of the SA-RBFNN which was introduced in Section 3.2 as the objective function, i.e., this objective function measures the difference between the predicted and true values *y*’ of the SA-RBFNN, so that the fitness obtained according to the objective function is also the error generated by the prediction result, which is negatively correlated to the fitness, in line with the minimization convention. Besides, in order to ensure individual richness at the beginning of evolution, and at the same time speed up the convergence when the population tends to converge, so that the convergence reaches the global optimum, the adaptive coefficient will be multiplied on top of the first part, which is positively correlated to the degree of evolution, as shown in Step 2 of Figure 2.

- Step 3: Selecting individuals

Next, individuals with a higher fitness level are selected to form the next population based on their fitness level. The basic idea behind various selection algorithms is that individuals with a higher fitness level have a higher probability of being selected, while those with a lower fitness level have a lower probability of being selected. Commonly used selection algorithms include the roulette wheel method, league selection method and elitist preservation strategy [35]. In order to solve the problems of local optimality and poor convergence, this paper will use the elitist preservation strategy for individual selection, as shown in Step 3 of Figure 2.

- Step 4: Crossover and Mutation operations

Crossover operation is a process whereby two mutually paired chromosomes exchange some of their genes with each other in some way, based on the probability of crossing over, to form two new individuals. Crossover operations play a key role in genetic algorithms and should produce as diverse an offspring as possible. Commonly used crossover methods are: single point crossover, two point crossover, uniform crossover, etc.

Mutation is the process of replacing some gene values in an individual’s coding string with other gene values to create a new individual based on the probability of mutation. Mutation in genetic algorithms is an auxiliary method for generating new individuals, which enhances the local search capability of the genetic algorithm while maintaining the diversity of the population. The common methods of variation are: binary mutation, Gaussian mutation, polynomial mutation, etc.

The probability of crossover and mutation operations is important and determines the convergence of the algorithm and the diversity of individuals. Two strategies are usually classified as having fixed probabilities and adaptive probabilities which is used adaptive strategy [36]. In the former case, the probability of the crossover mutation process is a fixed constant, while in the latter case the crossover mutation probability is adaptively changed according to the degree of evolution.

The crossover and mutation operations work together to complete the global and local search of the search space, while crossover and mutation play a decisive role in avoiding premature convergence. The crossover and variation methods in this paper choose two-point crossover and binary variation, respectively, and apply an adaptive probability strategy in probability selection, as shown in Step 4 of Figure 2.

- Step 5: Generating a new population

With the above four steps, one iteration is completed and a new parent population is generated for the next iteration, which will continue with the next cycle until the end point condition is reached and the best individual is obtained, as shown in Step 5 of Figure 2. 

### 2.3. Self-Adaptive RBFNN (SA-RBFNN)

As described in Section 2.1, there are two common methods for spread factors determination of RBFNN. However, the randomly selected spread factors of RI-RBFNN cannot ensure proper steep degree of activation functions, meanwhile empirical formulas driven determination of spread factors in EF-RBFNN cannot represent different distributions of clusters. In view of their drawbacks, a novel self-adaptive RBFNN (SA-RBFNN) is proposed to optimize the determination of spread factors via GA algorithm.

Actually, the spread factors value mainly refer to two aspects: the global distribution of dataset and the respective distribution of each cluster. Proper values of spread factors should be given as corresponding activation degree of activation function according to two aspects above. Qualitatively speaking, the spread factors are related to three parameters, i.e., the furthest distance between any two clusters *d*_max_, the number of clusters *L*, and dispersion degree of each cluster *p*. Larger *d*_max_ means the distribution of the dataset is sparse, and the clusters are different from each other obviously, thus the spread factor will be smaller so that the RBF will not be significantly activated by data belong to other clusters. Furthermore, larger *L* means that there are many independent clusters in dataset, and the possibility that the current sample belongs to different clusters is increasing, so the spread factor will be relative large meaning some RBFs might be activated at the same time. Finally, a smaller *p* means that the corresponding cluster is relative dense, and the RBF should be strongly activated by samples belong to this cluster, so the spread factor will be smaller. As stated, the spread factor is negatively correlated with *d*_max_ and positively correlated with *L* and *p*. Thus, the dispersion degree of each cluster can be calculated as follows.

(5) $$p_{k}=\frac{1}{n_{k}}\sum_{i=1}^{n_{k}}{\left(x_{k}^{i}-c_{k}\right)}^{2}$$

where, *p_k_* is the dispersion degree of the *k*th cluster, *n_k_* is sample number of the *k*th cluster, *xi k* is the *i*th sample, and *c_k_* is the center of the *k*th cluster. *p_k_* can be adaptive calculated according to different distribution of clustering data, and thus different clusters no longer share the same spread factor. As mentioned above, the parameters of *d*_max_, *L* and *p* are combined in Equation (6) to form a comprehensive formula for calculating the spread factors.

(6) $$\sigma_{n}=K*d_{\max}^{i}*L^{j}*p_{n}^{h}$$

where, *σ_n_* is the spread factor of the *n*th RBF, *K* is a constant coefficient, *d*_max_ is the largest distance between any two clusters, *L* is the number of clusters, and *p_n_* is the dispersion degree of the *n*th cluster. *i*, *j*, *h* are the exponential coefficients of the preceding parameters, respectively. Thus, GA algorithm is used to realize self-adaptive determination of multiple spread factors of RBFs according to Equation (6).

## 3. Overview of the Proposed Methodology

### 3.1. Hybrid Soft Sensor Model

**Figure 3:**
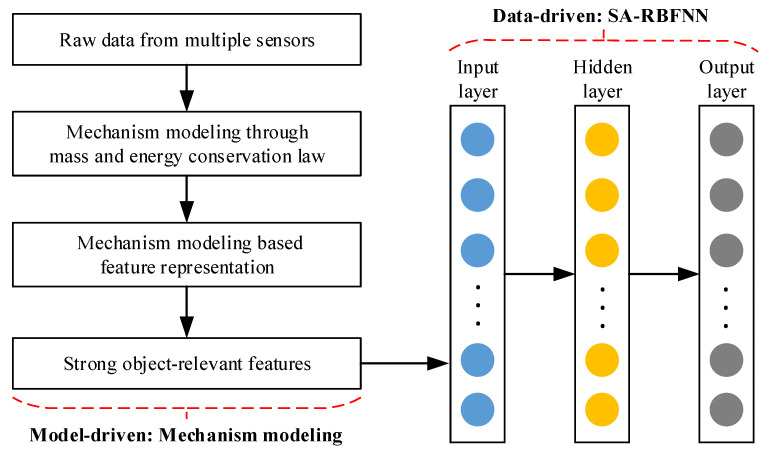
Architecture of the proposed hybrid soft sensor method.

Physical model based method and data-driven method are the common soft sensor methods in energy production. Due to the complex internal mechanism of thermal process in energy production, pure mechanism model always need certain assumptions that makes some deviations between the output of mechanism model and actual values. On the contrary, data-driven methods can be able to realize data modeling with no need of priori knowledge, but they excessively rely on the quality of training dataset with poor generalization. Hybrid soft sensor approach aims to combine priori physics knowledge and data-driven strategy, taking full advantages of both methods to improve the performance of soft sensor modeling. In this paper, taking coal mill ventilation (CMV) sensing in power plant as a case study, the architecture of the proposed hybrid soft sensor in this paper is shown in Figure 3.

As described in Figure 3, raw data are collected via hard sensors, and mechanism model of CMV is established through mass and energy conservation law. According to mechanism analysis, feature representation of CMV soft sensor is conducted, and strong relevant features of CMV are obtained for SA-RBFNN based soft sensing.

### 3.2. Model Evaluation

The goal of our work is providing convincing results for online soft sensor modeling in energy production process. Therefore, the accuracy and timeliness of soft sensor models are the main objects for model evaluation. The root mean square error (*RMSE*), which represents the error of predicted and actual value, is utilized to evaluate the fit degree of soft sensor model. The *RMSE* is formulated as follows:

(7) $$\;RMSE=\sqrt{\frac{1}{N}\sum_{i=1}^{N}d_{i}^{2}}$$

where $d_{i}=y-y^{\prime }$ is the error between the actual values $y^{\prime }$ and predict values y of the *i*th tested sample and *N* denotes the total number of testing dataset. When the *RMSE* is smaller, the better the model obtained.

Meanwhile, in order to estimate the computing efficiency of comparative soft sensor models, space complexity and time complexity of algorithms are calculated to verify that our method can meet the requirement of online soft sensor. Therein, number of hyper-parameters are used to represent the space complexity of soft sensor algorithms which is s expressed as *O_s_*, and the consuming time in training and testing processes indicate the time complexity of comparative models which is s expressed as *O_t_*.

## 4. Case Study

### 4.1. Motivation of CMV Soft Sensor

Air supply control system is one of the most important systems for boiler burning control systems in a power plant, and the accuracy and reliable sensing of CMV is a vital to improve the air distribution and combustion efficiency. Under the influences of continuous high-speed scour of dusty ventilation, the CMV sensing results using traditional contact-type hard sensor will drift seriously over time, deviating larger from actual values. In this case, soft sensing of CMV become an effective approach to solve such difficulties. 

### 4.2. Mechanism Modeling of CMV

On account of the length of CMV pipeline is much larger than the radius of CMV pipeline and the speed of CMV is lower. The airflow in CMV pipeline can be considered as unitary steady ideal flow. According to Bernoulli equation, the formula of air pressure for any two points in CMV pipeline is described below.

(8) $$P_{1}=P_{2}+\frac{1}{2}\rho V^{2}$$

where, *P*_1_ and *P*_2_ are any two air pressure in CMV pipeline, *ρ* is the air density and *V* is the mean value of air flow velocity. Therein, the variate *V* can be described as Equations (9) and (10).

(9) $$V=\sqrt{\frac{2(P_{1}-P_{2})}{\rho }}=\sqrt{\frac{2△P}{\rho }}$$

(10) $$\rho =1.293\times \frac{273.15}{273.15+t}\times \frac{101325+P}{101325}$$

As stated, the computation formula of the air mass flow in pipeline can be described in Equation (11).

(11) $$Q_{m}=K\times A\times \rho \times V=K\times A\times \sqrt{\rho \times \Delta P}$$

In the case of ignoring the air leakage of CMV pipeline, the total amount of CMV equals to the sum of the hot and cold air ventilation. The formulas of hot and cold air ventilation *Q_h_*, *Q_c_* are shown as follows.

(12) $$Q_{h}=\sqrt{\left(P_{i}-P_{h}\right)}\times \frac{\rho \times \sqrt{T_{h}}}{\left(R_{h}+R_{hv}\right)}$$

(13) $$Q_{c}=\sqrt{\left(P_{i}-P_{c}\right)}\times \frac{\rho \times \sqrt{T_{c}}}{\left(R_{c}+R_{cv}\right)}$$

where, *P_i_* is the primary air pressure of coal mill, *P_h_* is the exit air pressure of air preheater, and *P_c_* is the exit air pressure of primary air fan. *ρ*(*T_h_*) is the hot air density, and *R_h_*, *R_hv_* are respectively the wind drag of hot air pipe and hot air valve. Besides, *ρ*(*T_c_*) is the cold air density, and *R_c_*, *R_cv_* are respectively the wind drag of cold air pipe and cold air valve. Therefore, the formula of CMV can be calculated as follows.

(14) $$Q_{i}=Q_{h}+Q_{c}=Q_{h}\times \frac{\left(T_{h}-T_{c}\right)}{\left(T_{i}-T_{c}\right)}=\sqrt{\left(P_{i}-P_{h}\right)}\times \frac{\rho \times \sqrt{T_{h}}}{R_{h}+R_{hv}}\times \frac{T_{h}-T_{c}}{T_{i}-T_{c}}$$

where, *T_h_* is the exit air temperature of preheater, *T_c_* is the exit air temperature of primary air fan, and *T_i_* is the primary air temperature of coal mill. According to the Equation (13), the precondition of CMV calculation is to know the relationship of the wind drag and opening of hot wind valve. However, due to the complex distribution of drag components, this relationship is difficult to realize quantitative description. CMV sensing via Equation (14) is unrealistic, but mechanism analysis above can guide effectively feature representation of CMV.

### 4.3. Parameters Setting of SA-RBFNN

RBFNN is a non-linear network that approximates any continuous value with arbitrary accuracy by using multiple radial basis neurons, and in this paper, we use a Gaussian kernel function as activation functions for the hidden layer nodes. Considering the fact that using the conventional Gaussian kernel function has the same degree of dispersion, i.e., spread factor, for each cluster, this situation is not optimal. So, we attempt to conclude a new formula for quickly determining the spread factor, capable of dynamically measuring the dispersion of different class clusters, in which the hyperparameters in the formula are determined by an improved genetic algorithm. In this case study, the setting of hyper-parameters for the three comparative RBFNN based models are shown in the following Table 1. From Table 1, we can see most of the hyper-parameters are the same, such as epochs, learning rate, batch size, and number of clustering centers etc. The difference of them mainly lies in the calculation of spread factors, of which the SA-RBFNN is self-adaptation comparing to the constant values of RI-RBFNN and EF-RBFNN. Actually, the core of GA based optimization is the self-adaptation of spread factors for RBFNN based soft sensor modeling.

Moreover, hyper-parameters of GA mainly include population size, chromosome length, the probability of performing crossover, the probability of mutation and maximum number of generations. Therein, population size, chromosome length and maximum number of generations are constants set based on experience, and the probability of performing crossover and the probability of mutation is adaptively adjusted after the initial value is set. Thus, the detailed inforamtion of GA hyper-parameters is shown in Table 2.

### 4.4. Dataset Description

The dataset used in case study is #3 unit of Jinjie power plant of China Energy Investment Company., which equipped with positive pressure direct blowing powder system, and rated load is 600 MWe. The coal pulverizing system (CPS) consists of total of six HP-863 coal mills, one of which is a spare mill in daily operation.

The samples are collected by means of manual measurement in April 2013, the accurate ventilation was measured at the pipe satisfying the measuring conditions, and the ventilation sensor was calibrated at the same time. Then the related parameters were also collected for the validation of the proposed soft sensor method in this paper. The dataset includes the main parameters of the pulverizing system, including coal feed, primary air temperature at grinding inlet, primary air pressure at grinding inlet, differential pressure between upper and lower of grinding bowl, coal mill current, primary air pressure at grinding outlet, primary air temperature at grinding outlet, negative pressure of furnace, raw coal temperature of coal hopper and load, as shown in Table 3. The dataset was collected from 9:00 to next day 13:00, and the first 24 h were used as the training data of soft sensor modeling, and the remaining 4 h were used as the testing dataset. According to the mechanism modeling of CMV in Section 4.2, the parameters below are selected as the inputs of SA-RBFNN based soft sensor model.

### 4.5. Results and Discussion

**Figure 4:**
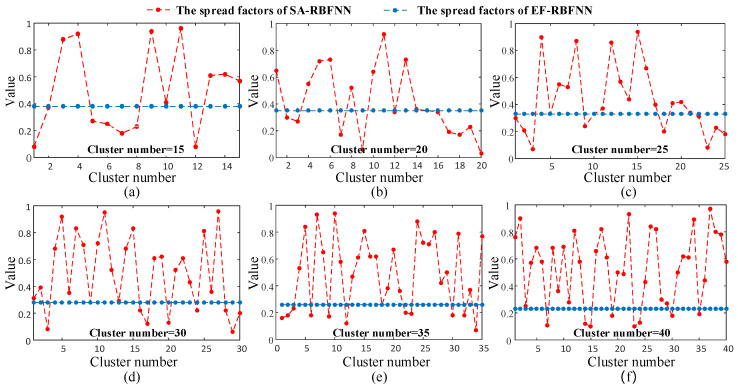
Spread factors of SA-RBFNN and EF-RBFNN under different cluster number setting.

As mentioned in Section 2.1, hyper-parameters of RBFNN are centers of RBF, spread factor σ of RBF and number of hidden layers. In this paper, the centers of RBF are determined via unsupervised clustering using k-means algorithm, and improved GA algorithm is adopted to optimize the determination of the spread factors for RBFNN. Figure 4 indicates the spread factors of RBFs using the proposed SA-RBFNN and the classical EF-RBFNN in the case study. It can be seen that the spread factors calculated by EF-RBFNN is invariable under different cluster numbers.

As described in Section 2.2, the optimization process of spread factors via GA algorithm is conducted according to the Equation (6). The constant coefficient *K*, and exponential coefficients *i*, *j*, *h* are calculated by least square method, which can find the optimal function representation by minimizing the errors of soft sensor. Therefore, a simple and effective new empirical formula is proposed, which is used for the self-adaptive determination of spread factors for RBFNN. The new empirical formula is given by Equation (15).

(15) $$\sigma =1.5086\times d_{\max}^{-2.9400}\times L^{0.2918}\times p^{0.0674}$$

As can be seen that, the values of *K*, *i*, *j* and *h* are respectively 1.5086, −2.9400, 0.2918, and 0.0674. The results confirm our previous analysis in Section 2.3 that spread factors are negatively correlated with the indicator *d*_max_ and positively correlated with the parameters *L* and *p*. And the spread factors calculated via SA-RBFNN can be self-adaptive for better performance of soft sensing, s show in Figure 4. As the utilization of GA algorithm, the hyper-parameters of the RBFNN can be dynamically adjusted, which can be seen in Figure 4. The spread factors of RBFNN models have good adaptive properties. Through the operation of clustering and the feature of local activation, the RBFNN provides good non-linear mapping properties for datasets with multiple distributions. With different numbers of clusters, our proposed empirical formula is able to provide a dynamic measure of the dispersion of each cluster in order to better reflect the global and local data distribution of the data without the need for a time-consuming and tedious optimization process. For other devices of the same type, the above empirical formula can be used to quickly and adaptively determine the distribution and dispersion of the current data clusters, which is general and convenient.

**Figure 5:**
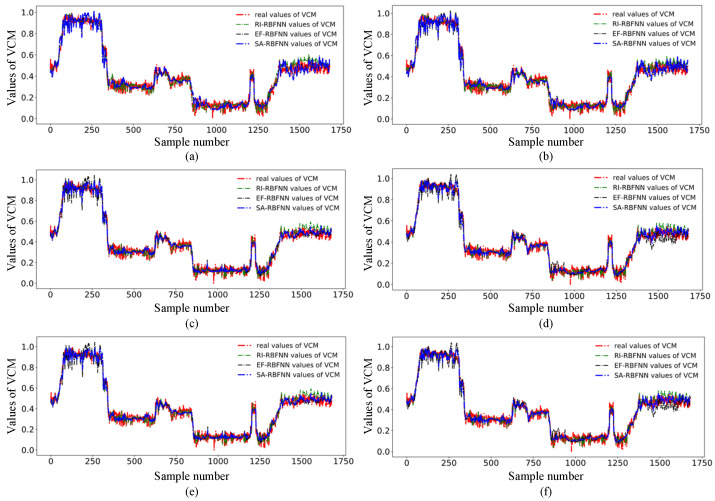
Soft sensing results of VCM using three RBFNN based methods under different cluster number (**a**) 15 clusters (**b**) 20 clusters (**c**) 25 clusters (**d**) 30 clusters (**e**) 35 clusters (**f**) 40 clusters.

To verify the effectiveness of the SA-RBFNN algorithm, VCM is calculated via RI-RBFNN, EF-RBFNN and SA-RBFNN, and the cluster number are set as 15, 20, 25, 30, 35, and 40 respectively. Therein the first 1445 points are training set, and the last 236 points are testing set. For a straightforward presentation and fit degree of the predicted results, the real values and soft sensing results of RI-RBFNN, EF-RBFNN and SA-RBFNN for VCM are shown in Figure 5.

In Figure 5, the red dashed lines are the real values, while the green, black and blue dashed lines are the predicted values from RI-RBFNN, EF-RBFNN and SA-RBFNN respectively. From the training processing, the predicted values for each type of RBFNN fluctuate within a reasonable range, but it is also clear that the other two models usually vary within a small range compared to SA-RBFNN, making it difficult for the predicted values to reflect the true variation and to truly reflect the subtle trends in the data within a range. From the testing part, the RI-RBFNN is unstable due to the random value of the spread factor, which varies considerably over a range of different clustering numbers, and the prediction error is larger, making the prediction results poorer. The EF-RBFNN has a traditional empirical formula for the spread factor, which has a particularly large range of variation in prediction at cluster numbers of 30 and 40, which is unstable and at the same time extremely poor in prediction, and a small range of variation at other cluster numbers, which basically fails to represent the details of the predicted values. So, it can be seen that the EF-RBFNN method with the classical empirical formula is very sensitive to the number of clusters and requires strong prior knowledge to determine the clustering of the data in advance. Compared with the other two methods, the proposed SA-RBFNN uses the feature of local activation to be able to adaptively represent the data distribution of each cluster and measure the degree of data dispersion, so that different clusters have different activation effects and accomplish more accurate prediction. The range of variation of the predicted values fluctuates within a reasonable range under various numbers of clusters. While the predicted value is accurately achieved, it is possible to represent the variation of the true value to the maximum extent possible.

**Figure 6:**
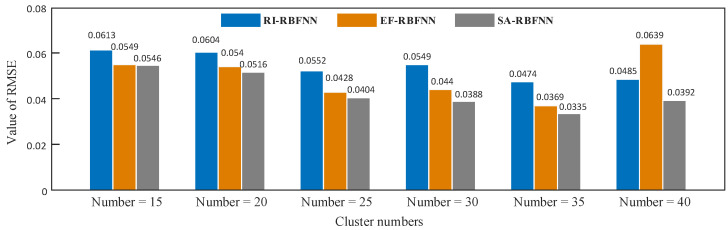
Comparison results of the comparative methods under different cluster numbers.

Furthermore, in order to statistically and numerically account for the error between the predicted and true values of each method, the root mean squared errors (*RMSE*) of comparative methods above on testing set are calculated and shown in Figure 6. Due to the random selection of the spread factor, the *RMSE*s of the RI-RBFNN are typically the highest for different numbers of clusters, while the value is known to be highly variable and unstable according to the rules of selection, which can directly affect the prediction accuracy of the true value. With the help of the empirical formula, the spread factor in the EF-RBFNN usually performs better than the RI-RBFNN in terms of prediction accuracy with a substantial improvement, implying a lower *RMSE* value. However, EF-RBFNN also suffers from a lack of stability, as can be seen from the result of a cluster number of 40. The soft sensor method based on SA-RBFNN achieved the best performance compared to RI-RBFNN and EF-RBFNN due to the local activation properties and advantages of the adaptive spread factor. Furthermore, the experiments also show that the minimum *RMSE* of SA-RBFNN is 0.0355 at the optimal cluster number 35, which improves its accuracy by 29.32% and 9.21% compared to RI-RBFNN and EF-RBFNN, respectively.

**Figure 7:**
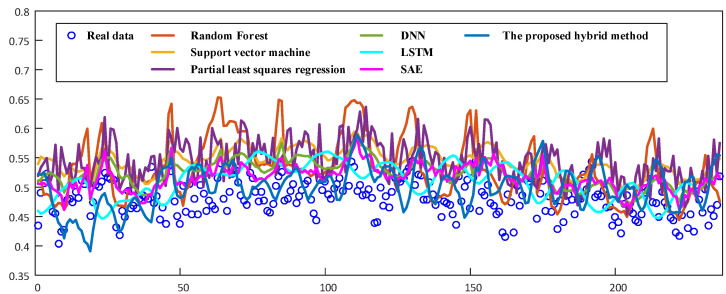
Soft sensing results of comparative models on testing dataset.

To further verify the advantages of the proposed methodology, comparative experiments between our soft sensor method and state-of-the-art models are conducted, and the comparison of model performance obtained is reported in terms of soft sensing accuracy and computation efficiency. In this experiments, three traditional machine learning and three deep learning based models, including random forest, SVM, partial least squares regression (PLS regression) [37], deep neural networks (DNN) [38], LSTM and SAE, are compared to verify the superiority of our proposed method. Figure 7 shows the soft sensor results of different models on testing dataset. 

The results in Figure 7 will be analyzed in two aspects, firstly our method will be compared with machine learning methods including random forest, support vector machine, partial least squares regression. In terms of prediction accuracy, there is a large gap with our proposed methods, which have poor prediction accuracy and large fluctuations. In terms of prediction trend, with more detailed features, it may have better ability to predict change trends. Compared to the machine learning methods, the deep learning methods have improved the prediction results, but the prediction magnitude is significantly smaller and the prediction trend is single and cyclical. Then, we evaluate the complexity of the proposed model in terms of space and time complexity, and compare the proposed model with that of other models below. It is important to note that we only compare the space complexity of the deep learning model. We express space complexity in terms of the number of network parameters and time complexity using one testing time. Our computer configuration was an Intel(R) Xeon(R) CPU E5-2620 v4 2.10GHz and a 12G NVIDIA TITAN X (Pascal) GPU with 32G of RAM. Table 4 shows the RMSE values and computational efficiency for all comparison models.

Experimental results in Table 2 demonstrate deep learning based soft sensor models perform better than traditional machine learning algorithms. Despite the shallow structure of SA-RBFNN, the proposed hybrid method is still superior to the comparative algorithms due to its nonlinear modeling ability of RBFNN and the optimal solution ability of GA algorithm. Furthermore, in view of online application scenario, we analyze the space complexity of ANN based models in terms of the number of network parameters, and detailed structures of them are designed first. DNN based model has 3-layer fully connected structure, and the number of 3-layer neurons are respectively 50, 10 and 10. LSTM based model has 2-layer LSTM structures and 2-layer fully connected structure, and the number of neurons are 32, 16, 2 and 1. SAE architecture has 4-layer fully connected structures, and has 30, 10, 10, 30 neurons in turn. Therefore, total parameter numbers of those models shown in Table 2 indicate our proposed method has the lowest space complexity. Finally, the consuming time of training and testing steps for all models is calculated. Comparative results indicate the training speed of our proposed method is inferior to the other machine learning algorithms, but is superior to deep learning models. The reason we analyzed is that SA-RBFNN model may consume extra time to get the optimal solution using GA algorithm. However, the proposed method performs better than all the other models in term of consuming time in testing process, which demonstrate the proposed method can meet the requirements of online soft sensor application.

## 5. Conclusions

In this paper, we propose a hybrid soft sensor method for condition monitoring of important process parameter in energy production process. The proposed methodology combines mechanism analysis and data-driven strategy, the former is adopted to finish the feature representation of objective parameter, and the latter is conducted by SA-RBFNN algorithm, which aims to integrate RBFNN and GA models to realize nonlinear soft sensor modeling and optimal solution calculation. 

We demonstrate that mechanism analysis is conducive to fully utilize the prior knowledge, and obtain the strong- relevant features of objective variate from high dimensionality of process data. And then RBFNN is set as a basic model for data-driven soft sensor modeling and GA algorithm is used to optimize the spread factors determination of RBFNN. Besides, relevant features obtained from mechanism analysis are inputted into SA-RBFNN based soft sensor model to provide an accuracy and reliable results.

Coal mill ventilation in one real power plant is taken as a case study to verify the effectiveness of our method. Results indicate SA-RBFNN method can obviously improve the performance of RBFNN based soft sensor model. Further investigation shows us the minimum *RMSE* of SA-RBFNN is 0.0355 at the optimal cluster number 35, which improves its accuracy by 29.32% and 9.21% compared to RI-RBFNN and EF-RBFNN, respectively. Therefore, the proposed methodology is superior to state-of-the-art soft sensor models in terms of sensing accuracy and computing efficiency. 

## Figures and Tables

**Table 1:** Hyper-parameters setting of comparative RBFNN based models.

Hyper-Parameter	RI-RBFNN	RI-RBFNN	SA-RBFNN
Epochs	100	100	100
Learning rate	0.01	0.01	0.01
Batch size	80	80	80
Number of clusters centers	15, 20, 25, 30, 35, 40	15, 20, 25, 30, 35, 40	15, 20, 25, 30, 35, 40
Number of input layer nodes	9	9	9
Number of output layer nodes	1	1	1
Number of hidden units	15, 20, 25, 30, 35, 40	15, 20, 25, 30, 35, 40	15, 20, 25, 30, 35, 40
Range of spread factors	0.16, 0.58, 0.36, 0.98, 0.47, 0.25	0.383, 0.333, 0.301, 0.274, 0.256, 0.245	Shown in Figure 4

**Table 2:** The hyper-parameters of GA algorithm.

Hyper-Parameter	Symbol	Values
Population size	*P*	100
Chromosome length	*L*	4
Probability of performing crossover	*P*_c_	0.9 (initial value)
Probability of mutation	*P*_m_	0.5/L (initial value)
Maximum number of generations	*N*	100

**Table 3:** Main parameters of coal mill in the case study of this paper.

Parameters	Descriptions	Units
*m*_c_	Coal quantity	t/h
*t*_c_	Raw coal temperature	°C
*t*_in_	Primary wind temperature at mill inlet	°C
*t*_out_	Primary wind temperature at mill outlet	°C
*p*_in_	Primary air pressure at mill inlet	kPa
*p*_out_	Primary wind pressure at mill outlet	kPa
∆*p*	Grinding bowl upper and lower pressure difference	kPa
*I*_m_	Coal mill current	A
*p*_b_	Furnace negative pressure	kPa
*D*	Power load	MWe

**Table 4:** Comparison results of space and time complexity for different models on testing dataset.

Model	RMSE	Space Complexity: *O_s_*	Time Complexity: *O_t_*	
		Total Parameters Number	Training Time (s per Sample)	Testing Time (s per Sample)
Random forest	0.0651	--	0.0425	0.0250
SVM	0.0695	--	0.0574	0.0018
PLS regression	0.0935	--	0.0496	0.0073
DNN	0.0496	1081	0.0961	0.0085
LSTM	0.0562	7525	0.1053	0.1845
SAE	0.0418	1051	0.0842	0.0165
SA-RBFNN	0.0335	595	0.0618	0.0016

## Data Availability

The data that support the findings of this study can be achieved when you contact me (songlei@csu.ac.cn).
